# Mitigating sub-synchronous oscillation using intelligent damping control of DFIG based on improved TD3 algorithm with knowledge fusion

**DOI:** 10.1038/s41598-024-65372-y

**Published:** 2024-06-26

**Authors:** Ge Liu, Jun Liu, Andong Liu

**Affiliations:** 1 College of Automation, Xi’an University of Technology, Xi’an, 710048 China; 2 Xi’an Aero-Engine Controls Technology Co., Ltd, Xi’an, 710072 China

**Keywords:** Sub-synchronous oscillation, Intelligent damping controller, S-TD3 algorithm, Adaptive output signal selection, Knowledge fusion, Energy science and technology, Engineering, Computer science

## Abstract

The occurrence of sub-synchronous oscillation (SSO) phenomenon in doubly-fed induction generators (DFIGs)-based wind turbines threatens the secure and stable operation of the power grid. Conventional sub-synchronous damping controllers encounter challenges in adapting to the dynamic operating conditions of power systems. This paper introduces an Intelligent Sub-Synchronous Damping Controller (I-SSDC) for DFIGs that integrates deep reinforcement learning (DRL) and knowledge to address the limitations of conventional methods for SSO mitigation. The initial step involves formulating a framework for I-SSDC using the improved twin delayed deep deterministic policy gradient (TD3) algorithm incorporating Softmax. Following this, a surrogate model is constructed, employing Weighted Linear Regression and regularization. This model is designed to identify the predominant influencing factors of SSO, focusing on the selection of the output signal (installation position) to optimize decision-making in I-SSDC. The objective is to enhance the controller’s environmental adaptability and interpretability. Moreover, knowledge and experience related to SSOs are integrated into agent training to improve the exploration efficiency of the agent. Case studies under various operating conditions of the test power system validate the efficacy of the proposed I-SSDC in suppressing SSOs.

## Introduction

### Motivation and incitement

Given the increasing global demand for green energy, wind power has emerged as a crucial player in power generation due to its abundant and renewable nature. Grid-connected wind power systems, particularly those utilizing doubly fed wind turbines with series capacitor compensation lines for transmission, are experiencing substantial growth within current power systems. However, this expansion brings about significant challenges, notably in the form of SSOs, which impact the system’s frequency and voltage stability^1,2^. For instance, an SSO event occurred at a wind farm in Texas in October 2009, primarily due to the interaction between power electronics related to Doubly Fed Induction Generators (DFIGs) and the adjacent series-compensated transmission line^3^. Similar SSO incidents have also posed challenges to power systems operations in Buffalo Ridge, Canada^4^, and Guyuan, China^5^. Operating conditions of the system can change at any time, and the frequency characteristics of triggered SSO may vary for different events in the same system. The characteristics of the triggered oscillation are influenced by factors such as wind speed, the number of online wind turbine generators (WTGs), and the degree of series compensation. Therefore, SSO can lead to undesirable consequences, including equipment damage, generation loss, and other power quality issues^6,7^. It is crucial to adopt a damping control strategy that can effectively mitigate SSO under the complexity of wind power systems and the dynamic operating environment^8^.

### Literature review

Currently, studies have explored strategies to suppress SSOs in DFIG-based wind farm grid-connected systems. The Sub-Synchronous Damping Controller (SSDC) method proves more suitable for engineering applications due to its low control cost, clear mechanism, and fast response. In Reference^9^, a comparison of additional damping control on the rotor-side converter (RSC) and grid-side converter (GSC) of DFIGs revealed superior mitigation performance on the RSC. However, due to the nonlinear relationship between controller parameters and system performance, it is challenging to determine the optimal parameters for this method. Reference^10^ introduced an improved sub-synchronous resonance damping controller and its corresponding control strategy, determining optimal gain coefficients using a particle swarm optimization algorithm. This method optimizes control parameters for different SSO frequencies but is only applicable for SSO suppression within a fixed frequency band. Traditional SSDC, based on the phase compensation principle, faces challenges in meeting oscillation suppression requirements under various operating conditions in grid-connected systems.

To overcome this challenge, various advanced control methods, including feedback linearization, sliding mode control, and robust control, have been proposed Reference^11^. Reference^12^ proposed a robust control method to suppress SSOs that is adaptable to changes in the output of multiple wind farms. Although robust control is applied to address changes in system states, quantitatively specifying the upper bound of uncertainties and disturbances a priori remains a formidable task. In Reference^13^, an innovative method for SSO analysis in wind power networks using linear optimal control was introduced. The approach involves developing a comprehensive mathematical model for wind turbines and applying linear optimal control theory to mitigate SSOs within wind power networks. Despite improvements in the design of SSDC, which simplifies parameter tuning, limitations persist due to the reliance on precise dynamic modeling of wind power systems, preventing online adaptive adjustments. Reference^14^ introduced an SSDC strategy employing an adaptive bandstop filter. However, the integration of spectral estimation and recursive least squares algorithm in this approach results in numerous design parameters, thereby elevating the complexity and design duration to some extent. Notably, all the aforementioned SSDC parameter designs adopt a model-based approach. Yet, such methods are susceptible to the influence of unmodeled dynamic links and other uncertain factors^15^, thereby potentially impacting the efficacy of damping control. Moreover, the oscillation frequency of the SSO is contingent upon the power grid environment, necessitating further validation of the aforementioned method’s efficacy in suppressing oscillations amidst frequency variations. In contrast, Reference^16^ identified the nonlinear dynamic characteristics and uncertainty issues inherent in DFIGs. To address these challenges, they proposed a novel robust disturbance observer fractional-order sliding mode controller, aimed at maximizing power extraction and enhancing fault ride-through capabilities. Additionally, Reference^17^ emphasized the significance of fault ride-through as a crucial performance metric for DFIGs. Their proposed solution involves enhancing this capability through a voltage compensator and introducing a dynamic voltage restorer to mitigate voltage fluctuations. Furthermore, Reference^18^ devised an effective Fractional-Order Sliding Mode Controller to precisely regulate the active and reactive power of DFIGs while mitigating system uncertainties and reducing chatter amplitude. Lastly, Reference^19^ presented a novel inertial control strategy enabling DFIGs to absorb or release kinetic energy via active power control, thereby facilitating participation in system frequency control.

In recent years, attention has shifted to Deep Reinforcement Learning (DRL) as a data-driven control method with robust nonlinear approximation capabilities and adaptability to complex environments^20^. DRL learns the mapping relationship from states to actions, enabling the acquisition of optimal strategies through continuous interaction with the environment and facilitating adaptive handling of variations in complex systems^21^. DRL-based damping controller design methods eliminate the need to study the system’s internal structure and mechanism or the controlled system’s mathematical model. They achieve rapid and effective online decision-making through offline learning and online applications. Reference^22^ employed the GrHDP algorithm to design a damping controller based on VSC-HVDC, effectively suppressing multi-mode low-frequency oscillations by adjusting the active power output of the inverter. The time delay caused by the transmission of remote signals will result in performance degradation of the controller. Reference^23^ utilized reinforcement learning technology to overcome communication delays and other nonlinear problems in wide-area damping control. Damping control methods based on reinforcement learning are often employed for transient oscillation suppression and low-frequency oscillation suppression within a small oscillation frequency range. However, they face challenges such as inadequate adaptation to operating conditions and the lack of real-time verification of control.

### Contribution and paper organization.

This paper introduces an innovative supplementary damping control method specifically designed for SSOs. The main contributions of this work are as follows:

- An Intelligent Sub-Synchronous Damping Controller (I-SSDC) based on the improved Twin Delayed Deep Deterministic Policy Gradient (TD3) algorithm is developed for DFIGs to mitigate SSOs. The inclusion of the softmax operation addresses the underestimation bias in the TD3 algorithm, enhancing its efficacy in the damping control process. A training method employing multiple samples is adopted, tailored for the suppression of time-varying and operationally diverse SSOs.
- A surrogate model is constructed using weighted linear regression and regularization, enabling the selection of the installation position for I-SSDC by explaining the regression model of participation factors. Compared with purely data-driven models, I-SSDC has better interpretability.
- Improvement strategies based on knowledge fusion are proposed to address the low training efficiency of current purely data-driven methods for intelligent agents. This strategy significantly accelerates the training convergence speed, which is beneficial for practical engineering applications.
- The performance of the I-SSDC is compared with traditional SSDC (T-SSDC) considering multiple operating conditions, including wind speed, active/reactive power output of wind farms, number of fans, and line series compensation degree.

This paper is organized as follows: “Methods Principle of Proposed I-SSDC” presents the system’s model with I-SSDC and the mitigation principle. Then, a data-driven SSO mitigation method using an intelligent sub-synchronous damping controller is proposed in the section “Design of I-SSDC based on improved TD3 algorithm with knowledge fusion”. The simulation results under multiple operating conditions are presented in section “Case study and experimental design”. Finally, major conclusions and potential directions for further investigation are given in Section “Conclusions”.

## Methods principle of proposed I-SSDC

**Figure 1:**
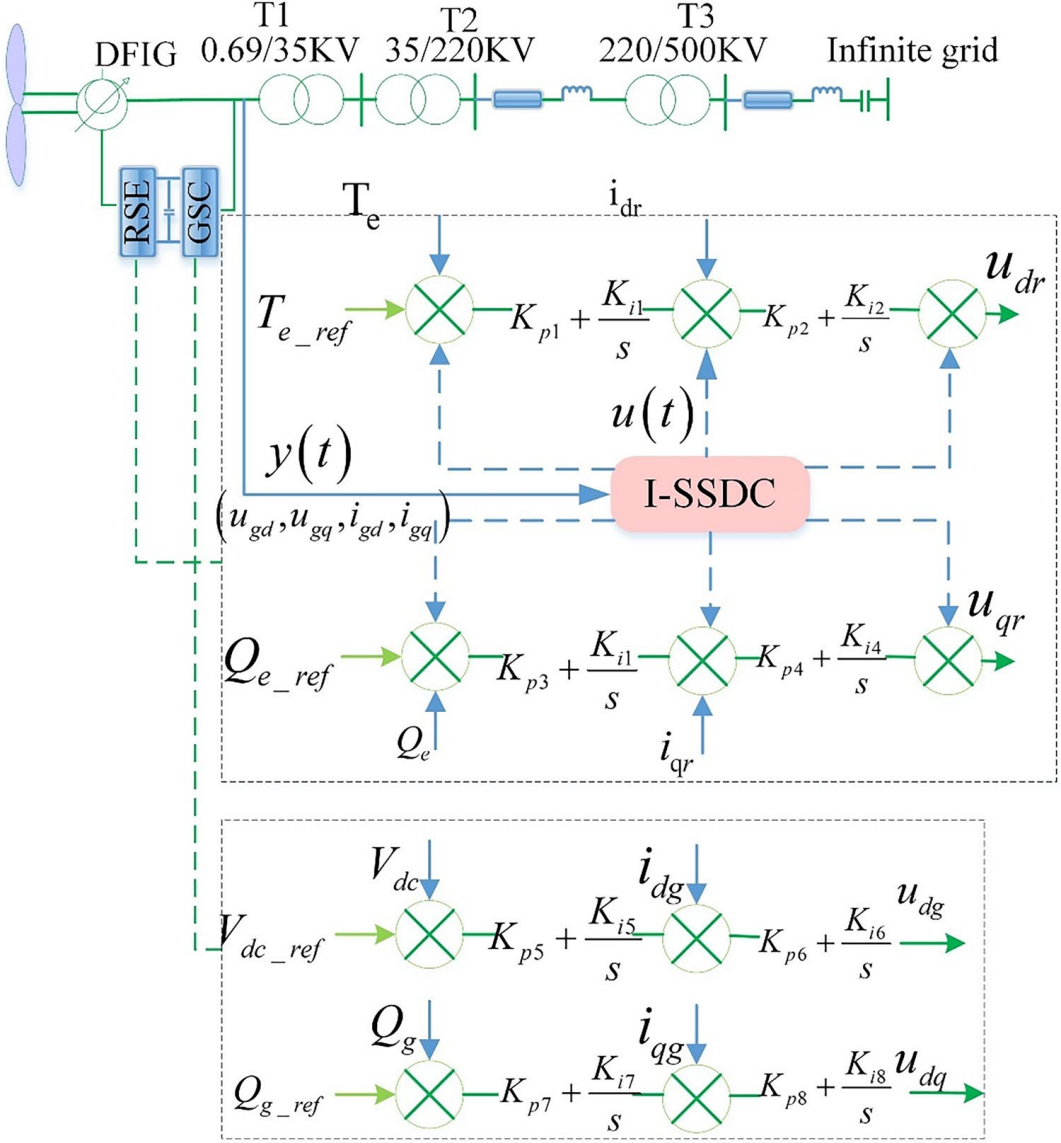
Equivalent model of I-SSDC suppression principle.

The equivalent model for SSO damping in DFIG-based wind farms with I-SSDC is shown in Fig. 1. The DFIG model comprises sections for wind turbines (WTs), DFIGs, inverter DCs, and rotor-side and grid-side converter controllers. The transmission system section, featuring series compensation, consists of a 220 kV line and a 500 kV line, with series capacitor compensation linked to the 500 kV line. Tables 1 to 3 in Appendix A meticulously delineate the parameters for each WTG, as well as the transmission line and transformer parameters.

### Modelling of the DFIG-based WT and its conversion system

The WT is the primary link of energy conversion in the wind power generation system. Mechanical output power and torque generated by the WT can be expressed as follows^24^.

1 $$P_{{{\text{mec}}}} = \frac{1}{2}\rho \pi R^{2} V_{w}^{3} c_{p} \left( {\lambda ,\beta } \right)$$

2 $$T_{mec} = \frac{1}{2}\rho \pi R^{2} V_{w}^{3} c_{p} \left( {\lambda ,\beta } \right)/\lambda$$

Here, *ρ* represents air density, *V*_*w*_ denotes wind speed, *R* signifies the radius of the WT rotor, *β* indicates the pitch angle in the variable pitch system, *λ* refers to the tip-speed ratio of the rotor, and *c*_*p*_ represents the WT’s power coefficient. *λ* and *c*_*p*_ can be expressed by the following equations:

3 $$\lambda = \frac{{Rw_{r} }}{{v_{w} }}$$

4 $$c_{p} \left( {\lambda ,\beta } \right) = 0.5176\left( {\frac{116}{{\lambda_{i} }} - 0.4\beta - 5} \right)e^{{\frac{ - 21}{{\lambda_{i} }}}} + 0.0068\lambda$$

5 $$\frac{1}{{\lambda_{i} }} = \frac{1}{\lambda + 0.08\beta } - \frac{0.035}{{\beta^{3} + 1}}$$

The DFIG is a complex system with high order, multivariable, nonlinearity, and strong coupling. In DFIG, the stator is directly connected to the grid, and its rotor is connected to the grid via a back-to-back converter for AC excitation. The stator and rotor voltage equations in the d–q reference frame can be illustrated as follows^25^:

6 $$u_{ds} = R_{s} i_{ds} + \frac{{d\psi_{ds} }}{dt} - w_{s} \psi_{qs}$$

7 $$u_{qs} = R_{s} i_{qs} + \frac{{d\psi_{qs} }}{dt} + w_{s} \psi_{ds}$$

8 $$u_{dr} = R_{r} i_{dr} + \frac{{d\psi_{dr} }}{dt} - \left( {w_{s} - w_{r} } \right)\psi_{qr}$$

9 $$u_{qr} = R_{r} i_{qr} + \frac{{d\psi_{qr} }}{dt} + \left( {w_{s} - w_{r} } \right)\psi_{dr}$$

Here, ${u}_{ds}$, ${u}_{qs}$, ${u}_{dr}$, and ${u}_{qr}$ represent the *d*-axis and *q*-axis components of the stator and rotor voltages, respectively; ${\omega }_{s}$ is the synchronous magnetic field rotation angular velocity; ${\omega }_{r}$ denotes the rotor angular velocity; $\psi_{ds}$, $\psi_{qs}$, $\psi_{dr}$, and $\psi_{qr}$ represent the *d*-axis and *q*-axis components of the stator and rotor magnetic fluxes.

The electromagnetic torque equation is as Eq. (10), where *n*_*p*_ is the number of pole pairs:

10 $$T_{e} = \frac{3}{2}n_{p} \left( {\psi_{qr} i_{dr} - \psi_{dr} i_{qr} } \right)$$

Rotor-side converter control consists of current inner loop control and power outer loop control. The reference value of the inner current loop depends on the maximum power point tracking (MPPT) curve (${T}_{e\_ref}$) and reactive power control of the outer power loop (${Q}_{e\_ref}$), respectively. The difference between the reference value and the rotor current feedback (${i}_{dr}$,${i}_{qr}$) is sent to the PI controller, and the ${u}_{dr}$ and ${u}_{qr}$ of the rotor voltage control are obtained. Through the conversion of d–q reference to a-b-c reference and PWM signal modulation, the power decoupling control of the rotor-side is realized.

The grid-side converter also employs double closed-loop decoupling control. The reference value of current inner loop control is obtained from the deviation of DC voltage (${V}_{dc}$) and reactive power (${Q}_{g}$) in outer loop control by the PI controller. The difference between the current reference value of the converter and the feedback quantity (${i}_{dg}$, ${i}_{qg}$) is input into the inner loop PI controller to obtain the voltage control signal (${u}_{dg}$, ${u}_{qr}$) of the converter on the grid-side.

### Mitigation principle of I-SSDC for DFIGs

The mitigation principle of the proposed I-SSDC for DFIGs resembles that of traditional SSDC, as illustrated in Fig. 1. Selecting electrical signals with significant sub-synchronous components from the measured electrical quantities of grid-connected nodes as the output signal $y(t)$ of the controlled system. This signal is then fed into the I-SSDC, producing a control output signal $u(t)$. SSOs in the DFIG-based wind farm grid-connected system primarily stem from the interaction between the controller of RSC and the series capacitor compensation circuit^26^. Consequently, the output signal $u(t)$ is integrated into the control loops of the RSC as the supplementary control signals, thereby generating damping torque/power and providing positive damping for the system. In contrast to traditional SSDC, this paper introduces I-SSDC to enhance adaptability to continuously changing environments and operational conditions. DRL, renowned for its learning and adjustment capabilities, proves advantageous for complex and dynamically changing systems like wind farms, as it does not rely on precise system models. TD3 is a DRL algorithm designed for deterministic strategies, making it well-suited for decision-making tasks involving continuous action spaces. Given that the environmental state variables of DFIG-based wind farms are continuous, I-SSDC adopts an improved TD3 algorithm based on measurement data to intelligently adjust damping control parameters through reinforcement learning, effectively mitigating SSOs.

### Principle of I-SSDC Based on DRL

**Figure 2:**
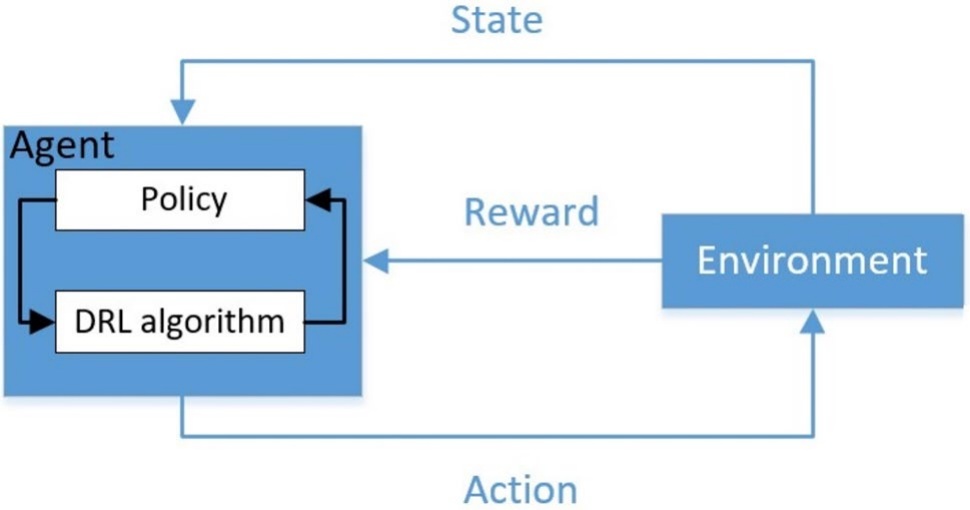
Interaction process between agent and environment.

Reinforcement learning involves a continuous interaction process between an agent and its environment to determine an optimal policy that maximizes the expected return. The key components include the environment, the agent, a set of states (s) representing the environment, a set of actions (a) representing the agent’s actions, and rewards (r) given to the agent. This interactive process is depicted in Fig. 2. In the context of I-SSDC based on DRL, the DFIG-based wind farm grid-connected system serves as the environment, with measured electrical quantities acting as the state for the agent. The agent determines the optimal action policy based on the state and received reward, i.e., the additional damping control signal.

1. The state, which is the perceptual information provided by the environment to the agent in the SSO suppression problem considered in this paper, is the input control signal to I-SSDC. Common input signals for additional damping controllers include rotor speed, terminal voltage of DFIGs, rotor current, etc. The input control signal for SSDC should possess characteristics that facilitate easy acquisition and fast transmission to minimize signal acquisition delays. The state set is defined as the oscillation amplitude of electrical quantities of grid-connected DFIG-based WTs:
  11 $${s}_{t}=[\Delta {u}_{gd}(t),\Delta {u}_{gq}(t),\Delta {i}_{gd}(t),\Delta {i}_{gq}(t)]$$
2. The action space comprises relevant decision variables in the optimization model. To suppress SSOs, the controller of RSC can be enhanced by adding additional damping control, thus injecting an extra damping control signal into the control system. The selected controller output signals include the inner and outer loop control output signals of the DFIG’s RSC. Dual-loop control is advantageous for suppressing SSOs. The action set can be defined as the injected additional damping control signals into the control system:
  12 $${a}_{t}=[{T}_{eAdd}(t),{Q}_{eAdd}(t)]\nu [{i}_{drAdd}(t),{i}_{qrAdd}(t)]\nu [{u}_{drAdd}(t),{u}_{qrAdd}(t)]$$
3. The reward function serves as a crucial driving signal for the intelligent agent to explore the optimal action strategy. The oscillation amplitudes of active and reactive power at the grid-connected node are critical for oscillation suppression. Therefore, the reward function for the agent is designed as Eq. (13):
  13 $${r}_{t}=-({\lambda }_{1}\Delta P(t)+{\lambda }_{2}\Delta {Q}_{e}(t))$$

Here, ${\lambda }_{1}$ and ${\lambda }_{2}$ require continuous experimentation and modification during training.

## Design of I-SSDC based on the improved TD3 algorithm with knowledge fusion

### Framework of I-SSDC

**Figure 3:**
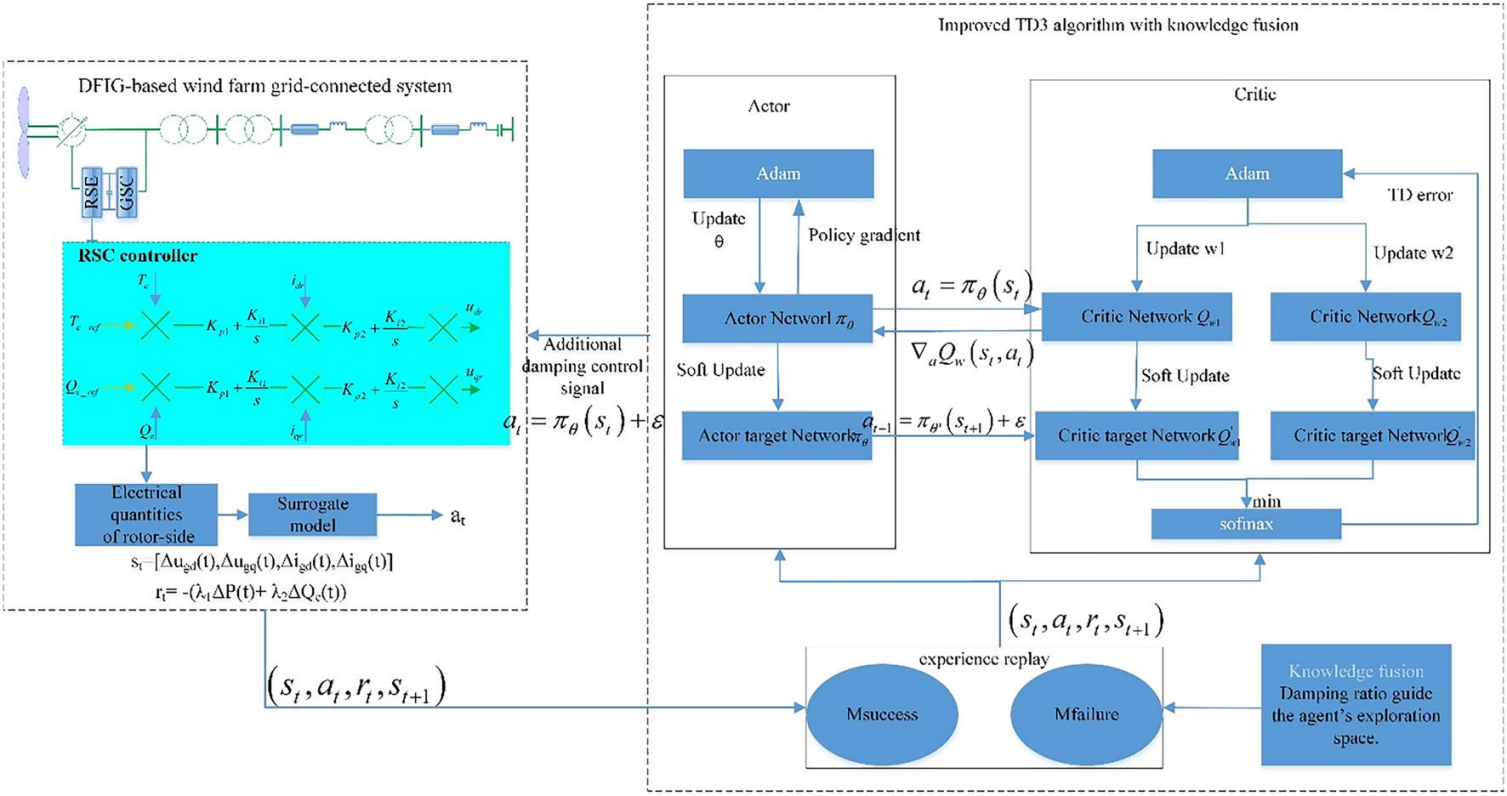
The Overall Schematic diagram of I-SSDC.

Figure 3 illustrates the overall schematic diagram of I-SSDC. The left side depicts the power system environment requiring additional damping, while the DRL agent on the right utilizes an S-TD3 algorithm, transforming the controller design into an MDP. The S-TD3 algorithm, an enhancement of TD3, incorporates the Softmax operation to control the gap between the value function and the optimal value, resulting in improved decision-making effects. A regression model is established between the electrical quantities of rotor-side control loops and contributing factors to adapt to the inhibitory effects of controllers in diverse scenarios. On this basis, a surrogate model extracts key characteristics from the electrical quantities to determine the optimal installation position for I-SSDC. The framework supports the integration of knowledge and experience related to the SSO parameter, restricting the agent’s exploration space. This is achieved by dividing the experience replay into successful experience replay and failure experience replay, followed by mixed sampling for agent training. In each episode, the agent attempts actions based on input states and performs a one-time domain simulation. The episode ends if the system’s oscillation amplitude is less than *ϵ*; otherwise, the search continues. Utilizing the samples generated by the interaction between the agent and the environment, the agent is trained to obtain the optimal action strategy for suppressing SSO through as few action attempts as possible.

### Adaptive output signal selection of I-SSDC based on a surrogate model

When deploying an additional damping controller to mitigate SSO, the controller’s output signal may impact its control efficacy. Moreover, the configuration of damping values often requires adjustments based on the operational state of the system^27,28^. Potential output signals available for the SSDC for control over RSC encompass the power control loops, d-axis and q-axis current control loops, and d-axis and q-axis voltage control loops. Employing traditional model-based observability and controllability indicators for generating output signals necessitates extensive online calculations. In this paper, the extraction of critical features from measured data of the rotor-side is employed to discern the optimal output for the controller, thereby enhancing the model’s performance and interpretability.

Exploring the relationship between electrical quantities and SSO is imperative to identify effective data features. The linearized state-space equations for the DFIG-based grid-connected system are presented below:

14 $$\frac{d}{dt}\Delta X=A\Delta X+B\Delta V$$

15 $$\Delta Y=C\Delta X+D\Delta V$$

Here, $\Delta X$ denotes the system’s state variables, ΔV represents the grid-side input voltage at the WT connection point, and $\Delta Y=[\Delta P, \Delta Q]$ signifies the active and reactive power injected by the WT into the power system. By employing the conventional modal analysis method^29^, the small disturbance stability of the system is characterized by the eigenvalues $\lambda$ of the system state matrix A. Each pair of complex conjugate eigenvalues corresponds to an oscillatory mode. The participation factors (${P}_{ki}$) are utilized to depict the influence of various system state variables on each oscillatory mode, as illustrated in Eq. (16).

16 $${P}_{ki}=\frac{\left|{\nu }_{ki}{w}_{ki}\right|}{\sum_{i=1}^{n} \left|{\nu }_{ki}{w}_{ki}\right|}$$

In Eq. (16), ${\nu }_{ki}$ and ${u}_{ki}$ represent the elements in the *k*-th row and *i*-th column of the left and right eigenvector matrices corresponding to the eigenvalue ${\lambda }_{i}$, respectively. Meanwhile, ${P}_{ki}$ signifies the correlation between the *i*-th mode (associated with the eigenvalue ${\lambda }_{i}$) and the *k*-th state variable. A larger ${P}_{ki}$ indicates a more significant influence of the state variable on this mode^30^.

With the characteristic matrix *A* of the closed-loop system given, the participation factors ${P}_{ki}$ can be determined, i.e., ${P}_{ki}=\partial \left(A\right)$, where $\partial \left(\cdot \right)$ denotes the mapping relationship function between the participation factor and the system characteristic matrix. The characteristic matrix A of the closed-loop system is dependent on the system’s operating point *M*. SSCI is primarily caused by the interaction between the RSC control and the series capacitor compensation circuit. When analyzing the SSCI problem, the influences of the grid-side converter, filters, DC capacitor, and phase-locked loop can be disregarded. Therefore, $M$ is expressed as $M=\left[{T}_{e}, {Q}_{e}, {i}_{dr}, {i}_{qr}, {u}_{dr}, {u}_{qr}\right]$, where ${T}_{e}$, ${Q}_{e}$, ${i}_{dr}$, ${i}_{qr}$, ${u}_{dr}$, and ${u}_{qr}$ represent the electrical quantities of the rotor-side control system. Consequently, the relationship between the participation factors and the system’s electrical quantities can be expressed as Eq. (17):

17 $${p}_{ki}=g(\partial (A))=g(\partial (h(M)))=l(M)$$

In Eq. (17), $g(\cdot )$ denotes the mapping relationship function between the participation factors and the system characteristic matrix; $h(\cdot )$ represents the mapping relationship function between the system characteristic matrix and the system operating point; $l(\cdot )$ signifies the mapping relationship function between the participation factors and the system operating point.

A regression model for the dominant participation factors under the primary SSO mode is established using a neural network method^31^ to estimate various operational scenarios. Building upon the regression model, this section employs a local surrogate model to extract critical features from multiple input characteristics under the currently studied sample, thereby determining the controller’s installation position selection.

The linear surrogate model $g(z)$ approximates the original model $f(x)$^32^, and its form is as Eq. (18).

18 $$g(z)={w}_{0}+\sum_{i=1}^{n} {w}_{i}{z}_{i}\approx f(x)$$

Here, $x$ represents the input of the sample, i.e., the electrical quantities of the rotor-side. $z$ represents $n$ important variables in $x$ that significantly impact SSO. By utilizing machine learning to obtain the parameters ${w}_{i}$ of the surrogate model $g(z)$ as interpretative results, the electrical quantity with the highest weight is chosen as the injection position for the output of I-SSDC.

Based on the input variables of the original model, sampling is performed with the training data of the surrogate model centered around the decision-making data (*x*_0_, *y*_0_). The estimated results of the original model’s sampling data are labeled. Constructing a linear model *g*′(*x*) based on weighted linear regression and L1 regularization, the model is trained using the sampled data. The important state variable *z* is selected from the sparsity of the parameters in *g*′(*x*), highlights the important state variables *z* that influence SSO. Since the regularization penalty in *g*’(*x*) is relatively strong, it leads to a larger parameter bias in the model solution. Therefore, further using *z* as input, a surrogate model *g*(*z*) is constructed using weighted linear regression and L2 regularization, training the model to make *g*(*z*) ≈ *f*(*x*). The objective function of the linear surrogate model is described as Equation (19):

19 $$\underset{w}{min} [L(w)+\sum_{i=1}^{n} \rho ({z}_{i})({y}_{i}-g({z}_{i}){)}^{2}]$$

In Equation (19), *L*(*w*) denotes the regularization term, and *ρ*(*z*_*i*_) refers to the weight coefficient of the sampled data. *L*(*w*) comprises L1 and L2 regularization terms, expressed as the follows:

20 $${L}_{1}(w)=\lambda \sum_{i=1}^{n} |{w}_{i}|$$

21 $${L}_{2}(w)=\lambda \sum_{i=1}^{n} {w}_{i}^{2}$$

The weighting coefficients of the samples can be determined using logistic regression, as shown Equation (22):

22 $$\rho \left({z}_{i}\right)={e}^{-{||{z}_{i}-{x}_{0}||}_{2}^{2}/2{\sigma }^{2}}$$

Here, the closer the sampled data is to ${x}_{0}$ during training, the larger the weight. $\sigma$ is a free parameter, and the smaller the value of $2\sigma$, the smaller the fitting neighborhood range of the linear surrogate model for $x$.

### S-TD3

The TD3 algorithm, structured as an Actor-Critic system as depicted in Fig. 3, engages in continuous interaction with the power system environment. This interaction acquires optimal values for the six neural network parameters, subsequently achieving an optimal configuration for the damping controller. This process is commonly referred to as offline training. The TD3 algorithm represents an enhancement of DDPG, introducing features such as clipped double-Q learning, delayed policy updates, and target policy smoothing^33^. Throughout the training process, the parameters $\theta$ and $\omega$ of the Actor network (${\pi }_{\theta }$) and critic network (${Q}_{w}$) are updated through gradient descent to minimize their respective loss functions.

The objective of the Actor network is to maximize the value function, utilizing a gradient descent approach to optimize the parameters $\theta$.

23 $$\left.{\nabla }_{\theta }J\left({\pi }_{\theta }\right)=\frac{1}{n}\sum_{i=1}^{n} {\left[{\nabla }_{a}{Q}_{w}\left({s}_{t},{a}_{t};w\right)\right|}_{\alpha =\pi \left({s}_{t}\right)}{\nabla }_{\theta }{\pi }_{\theta }\left({s}_{t}\right)\right]$$

In Eq. (23), *n* represents the number of training samples extracted from the experience replay, ${s}_{t}$ and ${a}_{t}$ denote the state and action at time $t$, respectively. Following the deterministic policy gradient, the parameters $\theta$ of ${\pi }_{\theta }$ are updated as $\theta =\theta +{\mu }_{\theta }{\nabla }_{\theta }J(\theta )$, where ${\mu }_{\theta }$ is the learning rate of the Actor network. Simultaneously, the parameters ${\theta }^{{{\prime}}}$ of the Actor target network are updated as ${\theta }^{{{\prime}}}=\tau \theta +(1-\tau ){\theta }^{{{\prime}}}$, with τ as the update coefficient.

The critic network optimizes parameter w by minimizing the loss function $Loss(w)$, defined as Eq. (24):

24 $$Loss\left(w\right)=\frac{1}{n}\sum_{i=1}^{n} {\left[{y}_{t}-{Q}_{w}\left({s}_{t},{a}_{t}\right)\right]}^{2}$$

Here, ${y}_{t}$ signifies the target *Q* value at time $t$.

The TD3 algorithm simultaneously learns two critic target networks (*Q*'_*w*1_ and *Q*'_*w*2_) and selects the minimum value for policy updates. While the TD3 algorithm incorporates a clipped double Q-learning mechanism to prevent *Q* value overestimation, it may introduce a low estimation bias on *Q* values, impacting performance. To effectively address these drawbacks, this paper introduces the S-TD3 algorithm, utilizing the Softmax function to estimate the value function. The softmax function can regulate the gap between the value function and the optimal value, reduce the frequency of obtaining local optimal solutions, and decrease the sensitivity of algorithm initialization parameters^34^. The target *Q* value (*y*_*t*_) can be expressed as:

25 $${y}_{t}=r\left({s}_{t},{a}_{t}\right)+\gamma softmax\left({Q}^{{{\prime}}}\left({s}_{t+1},{a}_{t+1}\right)\right)$$

26 $$softmax\left({Q}{\prime}\left({s}_{t+1},{a}_{t+1}\right)\right)=\frac{E\left[\frac{\text{exp}\left(\beta {Q}{\prime}\left({s}_{t+1},{a}_{t+1}\right)\right){Q}{\prime}\left({s}_{t+1},{a}_{t+1}\right)}{p\left({a}_{t+1}\right)}\right]}{E\left[\frac{\text{exp}\left(\beta {Q}{\prime}\left({s}_{t+1},{a}_{t+1}\right)\right)}{p\left({a}_{t+1}\right)}\right]}$$

27 $${Q}^{{{\prime}}}\left({s}_{t+1},{a}_{t+1}\right)=min\left({Q}_{w1}^{{{\prime}}},{Q}_{w2}^{{{\prime}}}\right)$$

Here, ${Q}^{{{\prime}}}\left({s}_{t+1},{a}_{t+1}\right)$ represents the minimum value of *Q*'_*w*_ obtained from the two critic target networks; *p*() is the probability density function of the Gaussian distribution; $\beta$ is the parameter in the softmax function; $\gamma$ is the reward discount factor.

${a}_{t+1}$ is calculated by the Actor target network ${\pi }_{\theta }^{{{\prime}}}$ and ${a}_{t+1}={\pi }_{\theta }^{{{\prime}}}({s}_{t+1})+\varepsilon$, where $\varepsilon$ is the added noise based on the normal distribution. The parameter *w* of ${Q}_{w}$ are updated according to the gradient rule, $w=w-{\mu }_{w}{\nabla }_{w}Loss(w)$, where ${\mu }_{w}$ is the learning rate of the critic network. The parameters ${w}^{{{\prime}}}$ of the critic target network are updated as ${w}^{{{\prime}}}=\tau w+\left(1-\tau \right){w}^{{{\prime}}}$.

### Agent training based on knowledge fusion

Knowledge fusion involves merging prior domain expertise with deep learning methodologies to enhance model performance and interpretability. This section presents an improved strategy that integrates relevant knowledge into the training of an agent within a data-driven approach, employing knowledge constraints to guide the agent’s exploration space.

The S-TD3 algorithm, a form of DRL, involves the agent interacting with a simulation environment, generating samples subsequently placed into an experience replay for training^35,36^. However, during the initial training phase, the agent is randomly initialized, posing a challenge for the agent to produce high-quality samples during interaction. This challenge results in a slow convergence of the agent toward an approximately optimal decision. To expedite the convergence speed of the algorithm, as depicted in Figure 3, knowledge rules related to SSO analysis are integrated into the decision-making process of the intelligent agent.

In instances of SSOs, the model of the actually collected measurement signals can be represented as Equation (28)^37^:

28 $$\text{x}\left(\text{n}\right)={A}_{1}\text{cos}\left(2\pi {f}_{1}n+{\phi }_{1}\right)+\sum_{i=1}^{t}{A}_{is}{e}^{{\xi }_{is}n}\text{cos}(2\pi {f}_{is}n+{\phi }_{is})$$

In Eq. (28), *x*(*n*) consists of the fundamental frequency component and the SSO component. ${A}_{1}$, ${f}_{1}$, and ${\phi }_{1}$ represent the amplitude, frequency, and phase of the fundamental frequency component, while ${A}_{is}$, ${f}_{is}$, and ${\phi }_{is}$ represent the amplitude, frequency, and phase of the SSO component, and ζ_*is*_ is the damping ratio of the SSO component.

The theory of SSO analysis in power systems indicates that the damping ratio quantitatively reflects the stability of the system. During SSOs, the damping ratio is negative, and the system’s stability diminishes as the damping ratio decreases. The objective of damping control is to maximize the damping ratio of the system, with the objective function defined as Equation (29):

29 $$\left\{\begin{array}{c}J=min{\xi }_{is}\\ max J\end{array}\right.$$

The Prony algorithm is employed to identify sub-synchronous frequency components at grid-connected nodes following SSCI. The computational steps of the Prony identification algorithm are detailed in the literature^38^, offering insights into SSO parameters such as amplitude, frequency, phase, and damping ratio.

This paper categorizes the experience replay into two sets: ${M}_{success}$ for successful experiences and ${M}_{failure}$ for failure experiences. Experiences from both successful and failure controllers are recorded using the identified damping ratio as a reference. Throughout the time-domain simulation process, the damping ratio at *a*_*t*_ is compared with that at *a*_*t-1*_. If *ζ*(*a*_*t*_) < *ζ*(*a*_*t-1*_), the controller at *a*_*t*_ is not conducive to stability, and *a*_*t*_ is assigned to the failure experience replay. Conversely, if *ζ*(*a*_*t*_) is greater than *ζ*(*a*_*t-1*_), the controller at *a*_*t*_ contributes to system stability, and *a*_*t*_ assigned to the success experience replay. The sampling ratios for the success and failure experience replays are as Eq. (30):

30 $$\left\{\begin{array}{c}{M}_{failure}=\beta m\\ {M}_{success}=(1-\beta )m\end{array}\right.$$

In Eq. (30), *m* represents the total number of training samples extracted from the experience replay; ${M}_{failure}$ and ${M}_{success}$ denote the sampling quantities of failure and success experience samples, respectively; $\beta$ is the sampling rate for the failure experience replay.

In ${M}_{success}$, the goal is to extract experiences of higher value, hence the implementation of prioritized experience replay. The data priority, denoted by $p$, is assessed through the TD error. The priority of the *k*-th sample is determined by Eq. (31):

31 $${p}_{k}=\left|{\delta }_{k}\right|=\left|{y}_{t}^{k}-\mathcal{Q}\left({s}_{t}^{k},{a}_{t}^{k}\right)\right|$$

The probability of sampling the *k*-th sample is determined by Equation (32):

32 $${p}^{{{\prime}}}(k)=\frac{{p}_{k}}{\sum_{n=1}^{{m}_{success}}{p}_{n}}$$

## Case study and experimental design

This paper focuses on the system model established in reference^39^ and constructs an equivalent system simulation model using the SIMULINK platform, as illustrated in Figure 1.

Each training episode lasts 5 seconds, with each step set to 0.0001 seconds in the configured improved TD3 agent. Diverse samples of operating conditions are selected for training to enhance the agent’s adaptability to the operating conditions of the DFIG-based wind farm grid-connected system. The system’s dynamic nature is captured by incorporating uncertain external parameters affecting SSOs, such as wind farm wind speed, control parameters, the number of WTs, active/reactive power output, series compensation degree, and transmission line parameters. The agent is trained using multiple samples generated by varying these parameters.

The configuration of the improved TD3 algorithm is detailed as follows: the critic network consists of three hidden layers with 128, 200, and 200 neurons, respectively. The Actor network comprises two hidden layers with 128 and 200 neurons each. Throughout the training process, hyperparameters are continuously fine-tuned, and the final selections are a critic network learning rate ${\mu }_{\omega }$ of 0.001, an Actor network learning rate ${\mu }_{\theta }$ of 0.0001, a successful experience replay (${M}_{success}$) capacity of 64, a failure experience replay (${M}_{failure}$) capacity of 64, a discount factor *γ* of 0.9, a soft update coefficient of 0.001, a sampling rate for the failure experience replay *β* of 0.8, and a noise standard deviation $\varepsilon$ of 0.3. The reward index coefficients are set as follows: *λ*_1_ is 0.7, and *λ*_2_ is 0.3. The surrogate model employs a kernel function with a parameter *σ* of 3, and the regularization coefficient $\lambda$ is set to 0.4.

## Results

### Performance of I-SSDC under various operating conditions

During the operation of the wind power transmission system, uncertain operating parameters undergo continuous random variations. It is imperative that the I-SSDC ensures real-time stability under these conditions.

**Figure 4:**
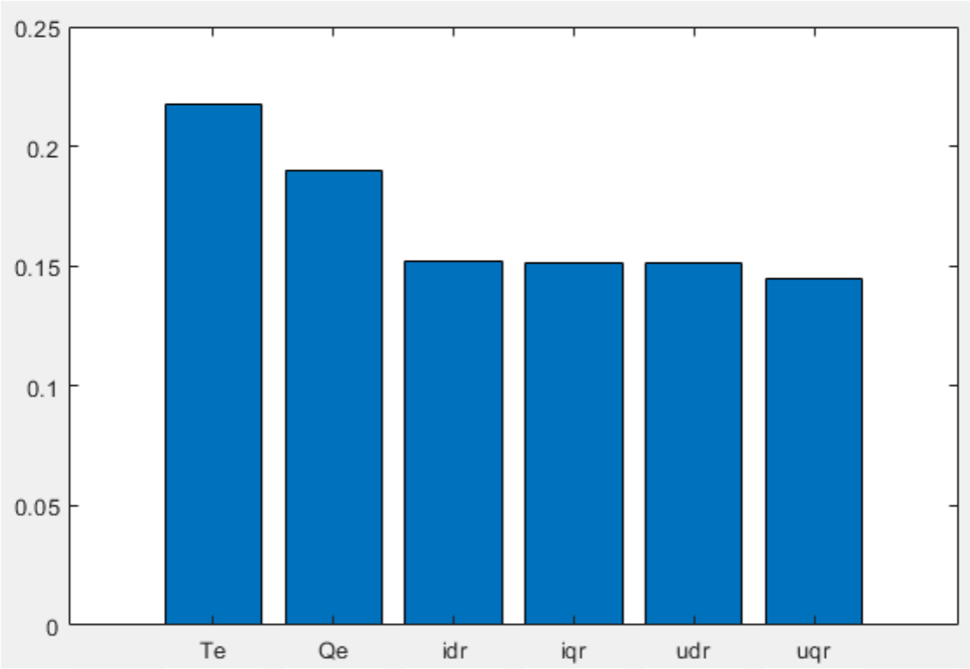
Influence weights of RSC control electrical quantities.

Utilizing the acquired system participation factors, a regression model is developed. A local surrogate model is fitted to the samples in its current operational neighborhood, offering insights into the significance of each feature under the prevailing conditions, as depicted in Figure 4. The sub-synchronous oscillation mode exhibits a pronounced correlation with the stator power of the DFIG-based WT. Consequently, the power loops of the RSC control are selected for sub-synchronous oscillation suppression.

**Figure 5:**
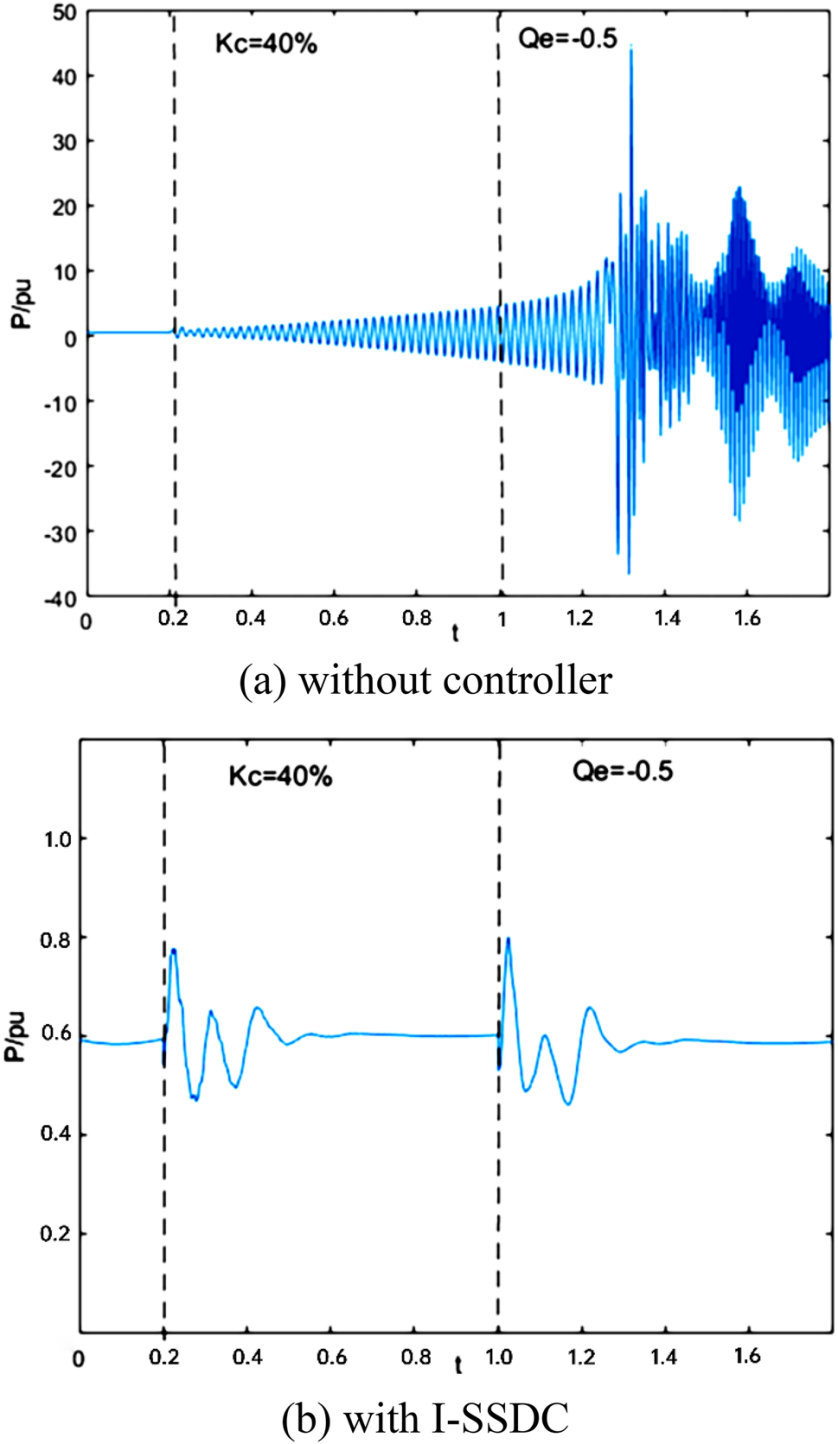
Suppression effect of the I-SSDC as the operating state changes over time.

To assess the real-time dynamic control efficacy of I-SSDC under varying system operating conditions, the initial operating state of the wind farm is characterized by a wind speed (v_wind_) of 9m/s, DFIG output active power (P) of 0.5 pu, and DFIG output reactive power (Q_e_) of 0. At t = 0.2 s, the series compensation degree (K_c_) is increased to 40%, and at t = 1 s, the reactive power (Q_e_) changes to − 0.5 pu. Figure 5 illustrates the time-domain waveform of the wind power plant’s active power output over time.

As depicted in Fig. 5a, active power oscillation disperses after 0.2 s due to the increased string complement. However, the oscillation amplitude is exacerbated by changes in output reactive power after 1 s, leading to the superposition of oscillation frequencies. Figure 5b illustrates that the amplitude of active power decreases substantially following the increase in string complement, owing to the involvement of the I-SSDC in the control process. This amplitude remains relatively stable after 0.45 s. With changes in output reactive power after 1 s, the I-SSDC adaptively updates according to system variations, maintaining its previous value once the oscillation subsides after 1.4 s. The oscillation frequency of the active power fluctuates after 1.2 s.

When the operating state undergoes a sudden change, the adaptive updating capability of the I-SSDC controller leads to a rapid decrease in the amplitude of the generator’s output active power oscillations. Once the oscillations are suppressed, the output active power returns to its previous value. This ensures the stable operation of the system under varying conditions. As the system operating point continues to change, the I-SSDC demonstrates excellent dynamic tracking control capability, successfully suppressing each disturbance-triggered SSCI consistently.

### Analysis of I-SSDC adaptability across a wide operating range

**Figure 6:**
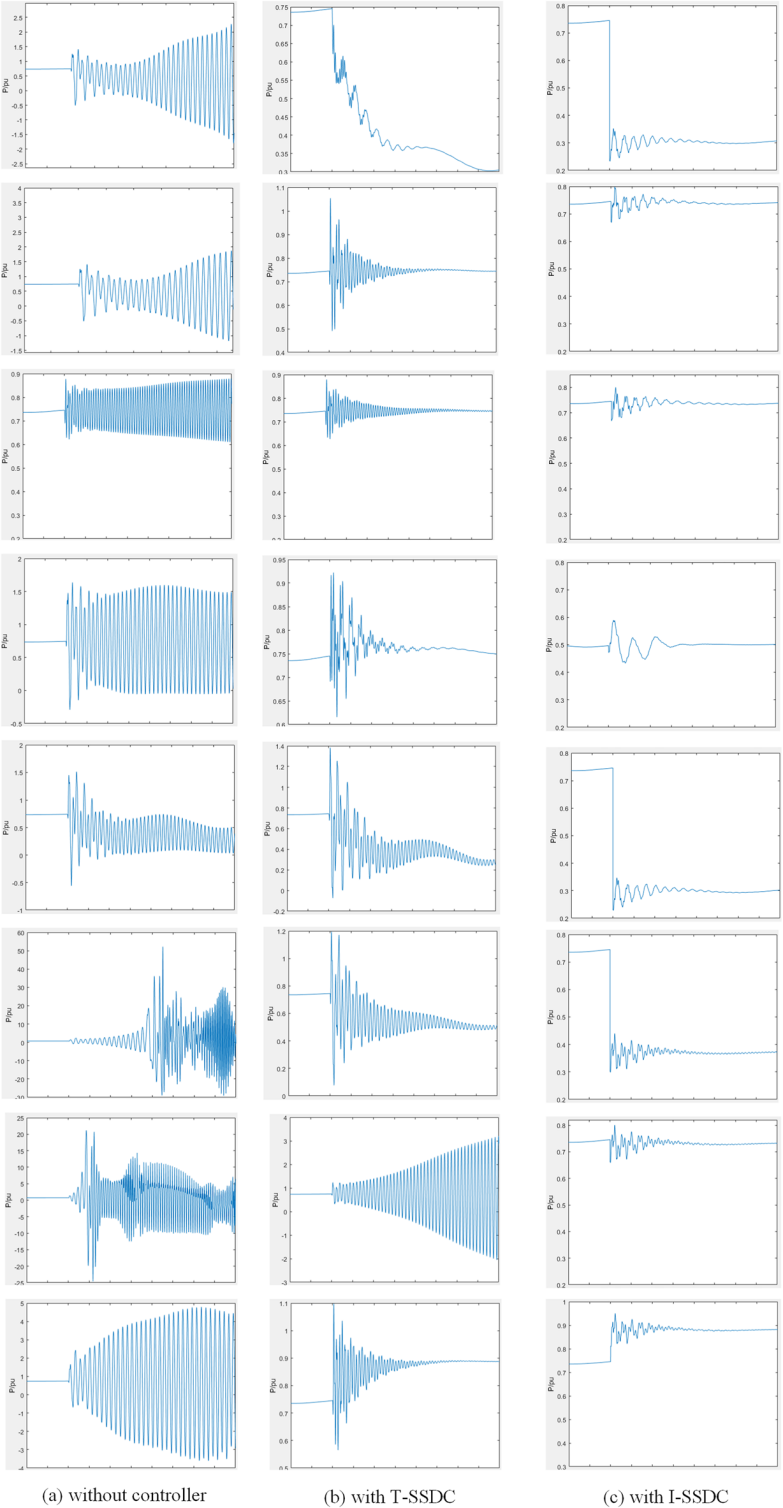
Suppression effect of the I-SSDC across a wide operating range.

To assess the enhanced adaptability of I-SSDC, a comparative analysis with T-SSDC) based on the phase compensation principle^9,10^ is conducted across a broad operational spectrum. This study comprehensively examines the adaptability of the proposed I-SSDC method compared to its traditional counterpart. The controller’s efficacy is evaluated under diverse operating conditions, including wind speed (V_wind_), active power of DFIG output (P), reactive power output of DFIG output (Q_e_), Controller parameter (I_d_-k_p_), and series compensation degree (K_c_). Establishing a stable state as the baseline operating point A serves as a reference for subsequent parameter adjustments, resulting in additional operating points B to I. Each operating point represents various levels of system stability. Points B to E modify a single operating parameter, points F to H alter multiple operating parameters concurrently, and point I serves as a non-training operating point. These points encompass a wide range of SSO frequencies due to substantial variations in operating parameters, posing a considerable challenge to the controller’s suppression capabilities. The operating conditions for each point are detailed in Table 1. The comparative evaluation between I-SSDC and T-SSDC at points B to I is presented in Fig. 6.

**Table 1:** Operating conditions of each operating point.

Operating point	V_wind_(m/s)	P(pu)	Q(pu)	I_d_-k_p_	Kc
A	11	0.74	0	0.01	10%
B	7	0.29	0	0.01	10%
C	11	0.74	0	0.01	90%
D	11	0.74	0	0.1	10%
E	11	0.74	− 0.5	0.01	10%
F	7	0.29	− 0.3	0.01	10%
G	9	0.50	0	0.01	50%
H	11	0.74	− 0.3	0.05	50%
I	12	0.88	− 0.3	0.01	50%

As shown in Fig. 6a, operating points B-E alter the wind speed, string complement degree, controller parameters, and output reactive power of the system, respectively. After 0.4 s, the system oscillates at a single frequency. Operating points F–H exhibit multiple superimposed oscillation frequencies due to changes in multiple operating parameters, resulting in higher oscillation amplitudes. From Fig. 6b, it is evident that after the system begins to oscillate, the oscillation amplitude at operating points B-E starts to decrease due to the involvement of the T-SSDC in the control. Significant reduction in oscillation amplitude is observed after 0.2 s of control participation, with the system returning to stability after 1.2 s. The speed of oscillation damping varies slightly among operation points B-E due to differing initial amplitudes. For operating points F–H, the system state changes after 0.4 s, with a gradual increase in the trend of oscillation amplitude reduction. However, even after 2 s, the oscillation is not entirely quelled, showing the worst suppression effect at operating point H. As depicted in Fig. 6c, the oscillations at operating points B-H subside after 0.6 s following the system state change, with a faster decrease in amplitude.

While T-SSDC succeeds in restoring system stability at operating points B to E, the suppression process is time-consuming, and there is a notable initial oscillation amplitude during control. However, at operating points F to G, SSCI is inadequately suppressed, indicating the limited adaptability of SSDC with fixed parameters.

I-SSDC adeptly suppresses SSO at operating points B to I, exhibiting faster convergence and less overshoot compared to T-SSDC. Notably, even at operating point I, the suppression strategy remains effective, showcasing the robustness of I-SSDC, even under non-training samples. In summary, a comprehensive comparative analysis is undertaken to demonstrate the superior adaptability of the proposed I-SSDC method compared to the effectiveness of T-SSDC, which is assessed across diverse operating environments.

## Conclusion

This paper addresses SSO in a DFIG-based wind farm grid-connected system by introducing an intelligent damping controller that amalgamates knowledge with improved TD3, ensuring the secure and stable operation of the power grid. Deep deterministic policies govern decisions regarding the control variable, particularly additional damping control, for action exploration. The Softmax operation enhances the accuracy of the trained model. A surrogate model, constructed using weighted linear regression, is employed. Relevant rules are synthesized by thoroughly examining the intricate and variable operating conditions. The selection principles for the output signal of I-SSDC are delineated, enhancing engineering practicality and interpretability. To further enhance the agent’s performance, introducing the key stability indicator, the damping ratio parameter, through knowledge experience-guided strategies improves decision-making frequency and reduces the number of decision iterations. Experimental results demonstrate the method’s robust adaptability to various operating conditions, effectively suppressing SSOs across variable and wide operating ranges. It holds promise as an advanced control strategy for ensuring the safe and efficient operation of DFIG generators.

Future research efforts will focus on refining the proposed DRL-based SSO damping control framework, integrating more specific operational experience to guide intelligent agent training, and leveraging increased computational power for validation in large-scale practical power grids.

### Supplementary Information

Supplementary Information.

## Data Availability

All data generated or analysed during this study are included in this published article [and its supplementary information files].

## Abbreviations

DFIG: Doubly fed induction generator
SSO: Sub-synchronous oscillation
I-SSDC: Intelligent sub-synchronous damping controller
SSDC: Sub-synchronous damping controller
DRL: Deep reinforcement learning
TD3: Twin delayed deep deterministic policy gradient
SSR: Static synchronous series compensation
WT: Wind turbine
WTG: Wind turbine generator
RSC: Rotor side converter
GSC: Grid side converter
MPPT: Maximum power point tracking
PI: Proportional-Integral
SSCI: Sub-synchronous control interaction
SSO: Sub-synchronous oscillation
VSC-HVDC: Voltage source converter high voltage direct current
RL: Reinforcement learning
MDP: Markov decision process
LSTM: Long short-term memory
Prony: Prony’s method
