# Supplementary material for: Mitigating sub-synchronous oscillation using intelligent damping control of DFIG based on improved TD3 algorithm with knowledge fusion
Source: Sci Rep. 2024 Jun 26;14:14692. doi: 10.1038/s41598-024-65372-y (PMC11208430; doi:10.1038/s41598-024-65372-y)
Supplement: Supplementary file 1 — Supplementary Information. [file 41598_2024_65372_MOESM1_ESM.docx]

# APPENDIX A

| Base capacity | 100MWA | DC link capacitor C | 14000μF |
| --- | --- | --- | --- |
| Rated voltage | 690V | DC link rated voltage | 1380V |
| X_ls_ | 0.09231 pu | Transformer ratio | 690V/35KV |
| X_lr_ | 0.09955 pu | Number of fans | 30–70 |
| X_M_ | 3.95279 pu | Active load | (0–0.3)pu |
| R_S_ | 0.00488 pu | reactive load | (-0.3–0)pu |
| R_r_ | 0.00549 pu | Wind speed | (7–11) m/s |

**Table 1.** Parameters of the aggregated DFIG in network system

| Parameters | Value | Parameters | Value |
| --- | --- | --- | --- |
| X_L_ | 0.5 pu | R_L_ | 0.02 pu |
| X_C_ at 50% compensation level | 64.8Ω | Series compensation level | 0.3–0.8 |

**Table 2.** Parameters of the network system

| Parameters | Value | Parameters | Value |
| --- | --- | --- | --- |
| GSC | | | |
| V_DC_-K_P_ | 2 | Q-K_P_ | 1 |
| V_DC_-K_i_ | 30 | Q-K_I_ | 30 |
| I_d_-k_p_ | 0.3 | I_d_-k_i_ | 10 |
| I_q_-k_p_ | 0.3 | I_q_-k_i_ | 10 |
| RSC | | | |
| Te-k_p_ | 0.5 | Q-k_p_ | 0.5 |
| Te-k_i_ | 2.5 | Q-k_i_ | 2.5 |
| I_d_-k_p_ | 0.01 | I_d_-k_i_ | 5 |
| I_q_-k_p_ | 0.01 | I_q_-k_i_ | 5 |

**Table 3.** Parameters of the converter controllers
